# Worth the Weight? Olanzapine Prescribing in Schizophrenia. A Review of Weight Gain and Other Cardiometabolic Side Effects of Olanzapine

**DOI:** 10.3389/fpsyt.2021.730769

**Published:** 2021-09-07

**Authors:** Ita Fitzgerald, Sarah O'Dwyer, Margaret Brooks, Laura Sahm, Erin Crowley, Ciara Ní Dhubhlaing

**Affiliations:** ^1^Pharmacy Department, St Patrick's University Hospital, Dublin, Ireland; ^2^School of Pharmacy, University College Cork, Cork, Ireland; ^3^Department of Medicine, St Patrick's University Hospital, Dublin, Ireland; ^4^Department of Pharmacy, Mercy University Hospital, Cork, Ireland; ^5^College of Mental Health Pharmacy (CMHP), Burgess Hill, United Kingdom

**Keywords:** metformin, antipsychotic-induced weight gain, schizophrenia, metabolic side effect, olanzapine

## Introduction

About 80% of patients who experience a first episode of psychosis (FEP) will have another within 5 years (1). In a large meta-analysis of randomized controlled trials (RCTs), 12 months of antipsychotic treatment reduced the rate of relapse from 61 to 26% in FEP, and from 64 to 27% in chronic cases (2). Long-term antipsychotic treatment has been associated with a decrease in mortality rates, compared to untreated cases (3). However, compared to the general population, those with schizophrenia still die, on average, 14.5 years earlier (3, 4). Most life years lost relate to poor physical health outcomes—specifically mortality related to cardiovascular disease (CVD) (4). Adverse cardiometabolic side effects of antipsychotics, particularly second-generation antipsychotics (SGA), represent an undeniable contributor to the burden of cardiovascular morbidity and mortality amongst this cohort (5). Olanzapine, a SGA with one of the most significant cardiometabolic side effect profiles (5, 6), continues to be one of the most popular antipsychotics prescribed in schizophrenia (7–9). This commentary will consider some of the most pertinent research assessing the efficacy of olanzapine in schizophrenia management; suggest potential reasons for incongruence observed between guideline recommendations and prescribing practices; and finally, make suggestions as to how olanzapine-induced weight gain—a side effect that often represents the beginning of a series of escalating cardiometabolic derangements–can be recognized and its effects ameliorated.

### Olanzapine and Weight Gain

Amongst those prescribed olanzapine early in psychosis management, 80% experience an increase of ≥7% of their baseline body weight (10, 11). With the exception of clozapine, whether short- or long-term anthropometric outcomes are being assessed, olanzapine is invariably listed as causing the most significant increases amongst users (6). This is not new information–the first review that aggregated data on weight changes following SGA use was published in 1999 (12). In a 2019 meta-analysis of 402 RCTs including data from 53,463 participants with multi-episode schizophrenia, olanzapine remained the antipsychotic most likely to induce clinically significant weight gain after clozapine and zotepine, the latter of which is not licensed in many countries (6). Antipsychotic-induced weight gain (AIWG) is a particularly important side effect, as it mediates cardiometabolic outcomes, including development of type 2 diabetes mellitus (T2DM) and subsequent CVD–the latter being responsible for approximately 60% of the excess mortality amongst those with schizophrenia previously highlighted (13). Aside from drastically reducing life expectancy, such high rates of physical comorbidity increases the personal, social and economic burden on mental illness across the lifespan. Significant weight gain may also influence outcomes related to mental health. AIWG is consistently listed as one of the most distressing side effects of antipsychotic treatment, frequently resulting in partial or complete antipsychotic non-adherence, and future reluctance to engage with treatment (14, 15). Furthermore, research is now emerging suggesting that obesity and metabolic syndrome may be independent predictors of relapse and re-hospitalization amongst those with a Severe Mental Illness (SMI) (16).

Due to its significant association with weight gain and other cardiometabolic abnormalities, since 2009 several international guidelines in Europe, the United States, Australia and New Zealand have advised against olanzapine's use as part of first-line psychosis management (17). However, today olanzapine continues to be one of the most popular antipsychotics, extensively prescribed in both FEP and chronic cases, across the spectrum of illness severity and in many jurisdictions (7–9). Considering the cardiometabolic adverse effects of olanzapine are well-documented, what might be the reasons underlying its popularity? Does its prevalent use reflect poor translation of evidence and subsequent guideline recommendations into clinical practice? Or does its popularity reflect an “availability bias”, whereby clinicians ascribe greater efficacy to olanzapine compared to other antipsychotics, simply based on their more familiar and frequent use of olanzapine? It is worth first assessing efficacy data supporting olanzapine, both in symptom reduction and relapse prevention.

### Efficacy of Olanzapine–Symptom Reduction

The best available evidence assessing this outcome can be found in the 2019 systematic review and meta-analysis of 402 RCTs referenced previously (6). Whilst some efficacy differences in symptom reduction were observed, differences were in general small, and uncertain in some cases, with few exceptions. Only amisulpride, olanzapine and risperidone were significantly more efficacious in reducing symptoms, compared to other antipsychotics (6). Previous analysis has shown that in the case of mild-moderate schizophrenia, the Number Needed to Treat (NNT) to produce significant symptom reduction was 10, and in moderate-severe cases, the NNT reduced to three (18). Similar results have been seen in the most recent meta-analyses of 8 RCTs where participants (n = 528) had a diagnosis of FEP (19). Such efficacy differences between antipsychotics–if identified consistently–become more clinically meaningful in the severely ill, where antipsychotics will be more effective at reducing symptoms. Additionally, the trade-off between therapeutic benefit and the burden of cardiometabolic side effects will be less. Thus, consensus remains as it has for many years–initial antipsychotic choice should primarily be driven by informed patient preference led by knowledge of the differing side effect profiles (20–24).

### Efficacy of Olanzapine–Relapse Prevention

In comparison to availability of data on olanzapine efficacy in acute phase management, similar data on outcomes addressing remission and relapse rates is lacking. When compared to placebo, a 2012 meta-analysis of RCTs with 6,493 participants found that there was not enough data specific to olanzapine to allow meaningful synthesis and subsequent comparison of outcomes (18). When compared to other antipsychotics, a meta-analysis of 23 RCTs with 4,504 participants found olanzapine to be no more effective at reducing relapse rates over a mean duration of 62 weeks. When data on first- and second-generation agents were pooled, results showed SGA to be more effective than first-generation agents (FGA), although the NNT was 17 (25). Observational studies in this area are useful when assessing longer-term outcomes and participants that are more representative of those presenting in clinical practice. A 20-year prospective cohort study assessed relapse rates of 8,719 participants with FEP and 62,250 with chronic schizophrenia and found that, over a median of 14.1 years, participants receiving olanzapine long-acting injection were the least likely to be re-admitted to a psychiatric hospital or to experience all-cause re-hospitalization (3). However, the effect size was comparable to other long-acting FGA and SGA, and to clozapine in chronic cases. The overlapping confidence intervals between point estimates meant that efficacy differences favoring olanzapine were no longer considered significant for many comparisons (3). Despite the lack of supporting evidence, many clinicians prescribe olanzapine in the first instance for acute management of FEP (7–9). Its sedative and anxiolytic properties likely influence this preference (9). However, acute agitation can be effectively managed with alternative medications. Given olanzapine's common and often early-onset cardiometabolic adverse effects (6), combined with an understandable reluctance to change antipsychotic after a period of stability, often these temporary sedative properties become distinctly less advantageous.

### Management of Olanzapine-Induced Weight Gain

In cases where olanzapine has been prescribed in the first instance and found to be effective, or where commencing treatment justified, for example due to previous failed trials of other antipsychotics, proactive management of cardiometabolic side effects, in particular weight management, should be an essential component of a holistic care. Amongst those presenting with FEP, the standardized mortality ratio is already raised compared to age- and sex-matched controls, even amongst those who are antipsychotic-naïve (26, 27). As the bulk of olanzapine-induced weight gain typically occurs within the first months of treatment (6, 11), early intervention strategies to improve cardiometabolic outcomes are imperative. To date many interventions have focused on the patient's role in improving their own cardiometabolic health. In contrast the development of strategic clinical care pathways to address this issue within psychiatric services, has been neglected (5, 28). Currently across the UK and Ireland, there is no care pathway or evidence-based intervention that is applied consistently and systematically when managing AIWG (28). Current practice is typically based on a “one-size-fits all” approach that seemingly mimics the hierarchical model applied in the general population (19–23, 27). This model does not adequately account for the disproportionate number of risk factors this cohort face for becoming obese, or the unique challenges patients must navigate due to the disease and its treatment (27). These challenges include expecting patients, often with prominent negative symptomatology, for example apathy, amotivation and low mood, to adhere to the rather simplistic and generic narrative regarding modifiable lifestyle factors. Furthermore, the current model considers all antipsychotics to present with similar risk of inducing weight gain, and discounts the genetic, clinical and demographic risk factors that lead to substantial interindividual variability in weight outcomes following antipsychotic initiation (12). Although a relatively unaddressed area, a review of the current standard of practice has left patients feeling unheard, and highlighted the disparity of values amongst patients, clinicians, and policy makers when it comes to managing AIWG (28, 29).

## Discussion

### Pharmacological Management of Olanzapine-Induced Weight Gain

Pharmacological management of weight gain, specifically that induced by olanzapine and other high-risk antipsychotic should be prioritized, particularly in cases where global functioning is poor. Currently metformin represents the intervention with the largest and most consistent evidence base amongst pharmacological management options and has the potential to significantly reduce the burden of AIWG when used early in antipsychotic treatment. Metformin treatment also has the ability to simultaneously modify several cardiometabolic parameters amongst those with schizophrenia (30). Yet in practice, its use remains inconsistent and underutilized (20, 28). Most recommendations addressing AIWG management recommend only considering pharmacological management once other interventions have failed, including diet and lifestyle interventions, and switching antipsychotics to a lower-risk agent (20–22). Since publication of the last of these guidelines in 2018 (21), two new meta-analyses of RCTs have been published. Both demonstrate a general lack of evidence to support switching antipsychotics as an effective means of attenuating AIWG, primarily due to lack of robust evidence (30, 31). Lifestyle interventions associated with the largest effective sizes are those tailored to the individual and delivered by trained nutritional and exercise professionals (30). Whilst ideal, lack of resources, including time constraints, inadequate training, and financial support prevent the widespread use of such interventions in clinical settings (28). Metformin treatment has been associated with similar effect sizes to that of “group lifestyle” interventions—the typical method of delivery of diet and lifestyle advice (30).

Whilst access to lifestyle interventions should be prioritized, guidelines and clinical policies need to consider the specific needs of this patient cohort and the context in which such interventions are typically delivered (32). In our opinion, based on the evidence available, use of metformin should be explicitly considered as part of an early, first-line intervention strategy to proactively manage AIWG. It's use is associated with low acquisition cost and associated resource use, very rare likelihood of causing catastrophic harm (30, 33), and is available to a much more widespread and socioeconomically diverse group in a sustainable manner compared to lifestyle interventions (30). Comparing the adverse effects of AIWG on both physical and mental health, negative associations with metformin, primarily polypharmacy and associated risks, become relatively less significant. On balance, co-prescription of metformin to a much wider range of patients prescribed olanzapine, and indeed many other antipsychotics, should be considered as a proactive early intervention to effectively manage AIWG.

Where metformin is not found to be effective, or in cases where clinical presentation or patient preference dictate alternative options be considered, several other pharmacological adjuncts with demonstrable efficacy in managing AIWG are available for consideration following clinically significant AIWG (30). However, compared to metformin, there are many other clinical considerations that need to be considered as part of the risk-benefit assessment. In a 2019 meta-review examining the comparative efficacy of all pharmacological adjuncts studied in reducing AIWG, topiramate was associated with a similar effect size in weight reduction (Standardized mean difference SMD −0.72, 95% CI −1.56 to −0.33) (*P* < 0.001), to metformin (SMD−0.53, 95% CI −0.69 to −0.38) (*P* < 0.001), although the point estimate was associated with considerable uncertainty, ranging from a small to large effect size. Topiramate is considered a teratogen, is contraindication in females of childbearing potential and is also commonly associated with a range of psychiatric side effects, including depression, insomnia, and less commonly suicidal ideation (34). Both considerations significantly limit use amongst psychiatric cohorts. Aripiprazole augmentation has also been associated with similar effect sizes in weight reduction (SMD −0.73, 95% CI −0.97 to −0.48) (*P* < 0.001), when compared with metformin or topiramate. Side effects commonly associated with aripiprazole, including akathisia and agitation, often limit dose titration to higher doses of 15 mg—the most commonly dose applied in AIWG management (30). Combined use of 15 mg of aripiprazole with olanzapine doses >10 mg would also lead to the prescription of high-dose antipsychotic therapy and the associated risks, including medico-legal responsibilities. Treatment with a Glucagon-Like Peptide 1 (GLP-1) receptor agonist e.g., liraglutide, is now emerging as a promising intervention to manage AIWG due to potential benefits on modifying cardiovascular morbidity and mortality through improvement in weight and blood glucose outcomes, as studied extensively in the T2DM population (35). Studies replicating these outcomes have yet to be conducted amongst those with schizophrenia. In the 2019 meta-review, short-term treatment with a GLP-1 receptor agonist was associated with a medium effect size (SMD −0.44, 95% CI −0.60 to −0.28) (*P* < 0.001). Other pharmacological adjuncts studied included amantadine, which was associated with a small effect size on weight reduction (SMD −0.30, 95% CI −0.57 to −0.03 (*P* < 0.05). All other adjunctive treatments included were associated with negligible or non-significant differences (30).

### Moving Forward

Whilst research on the adverse physical health effects of olanzapine and comparative data demonstrating its lack of superiority over alternative antipsychotics has been accruing over two decades (6, 12), subsequent change in prescribing practices, has not occurred at the same speed (7–9). To tackle such a complex problem, future management approaches will need to be multifaceted and scalable. One area deserving specific attention is an evaluation of methods shown to improve evidence-based antipsychotic prescribing. Different antipsychotics present with significantly different cardiometabolic risk profiles (6) and thus, optimizing their use to improve physical health outcomes in the absence of deterioration in mental health is an area of large potential impact. In cases where high-risk antipsychotics are required, standardized, systematic, and potentially risk-stratified pathways to manage AIWG are urgently needed as its presence often signifies the beginning of a series of adverse metabolic sequalae. As there is significant interindividual variability in the extent of total weight gained following antipsychotic initiation, future management algorithms would ideally incorporate results of quality research on identifying those who are likely to experience the most significant weight gain early in antipsychotic treatment. Any clinical care pathway developed also needs to reflect the values and preferences of patients affected by AIWG regarding preferred management approaches and goals of treatment; an aspect that has not been adequately researched to date. Success of such clinical care pathways will rely on a culture change globally, to shift focus from solely the individual role of the patient in managing AIWG, and toward clinicians and systems adopting and resourcing proactive, early interventional strategies.

## Author Contributions

IF, SO'D, MB, and CN contributed to the design of this opinion piece in collaboration with EC and LS. IF wrote the manuscript with input from SO'D, MB, CN, LS, and EC. All authors approved the final submitted version and agreed to be accountable for the content of the work.

## Conflict of Interest

The authors declare that the research was conducted in the absence of any commercial or financial relationships that could be construed as a potential conflict of interest.

## Publisher's Note

All claims expressed in this article are solely those of the authors and do not necessarily represent those of their affiliated organizations, or those of the publisher, the editors and the reviewers. Any product that may be evaluated in this article, or claim that may be made by its manufacturer, is not guaranteed or endorsed by the publisher.
